# The League of Mercy

**Published:** 1910-12-24

**Authors:** 


### THE LEAGUE OF MERCY.

### ANNUAL MEETING OF PRESIDENTS.

The annual meeting of Presidents of the League was held in the Board Room, Middlesex Hospital, on Monday, December 19, under the chairmanship of the Rt. Hon. the Lord Farquhar, G.C.V.O.

The Secretary (Colonel F. J. Kempster, D.S.O.) read the minutes of the last annual meeting, which were duly confirmed and signed.

### A Letter from King George.

The Chairman said it was his first duty to read a letter which had been sent by Sir Arthur Bigge by command of his Majesty the King in the following words: "In commanding me to make this communication to you as Chairman of the meeting of the Presidents of the League of Mercy, the King is more than ever reminded of the great loss which the League has sustained by the death of his beloved father, the founder and originator of the League. For the last ten years the King and Queen, as Grand President and Lady Grand President, have closely watched and personally supervised the work and extension of this organisation, and their Majesties feel sure that the Presidents and Lady Presidents will continue to render their best assistance to their successors, the Prince and Princess Alexander of Teck. As Patron, the King now takes this opportunity of assuring the Executive Officers, Presidents and Lady Presidents, as well as the Vice-Presidents, that his interest in their good work continues unabated; and his Majesty earnestly trusts that every success may attend their efforts to relieve the suffering of their less fortunate fellow-creatures." (Applause.)

### The Chairman's Speech.

Lord Farquhar was sure everyone present most cordially sympathised in every word of that letter, and he felt equally assured that everyone would do his and her utmost to work hard and meet the wishes which were expressed in the letter of their Majesties. That would be the first wish of everyone there. And the League of Mercy shared deeply with every other charitable institution in this country, and indeed with the great mass of the people, in mourning the heavy loss which was sustained by the death of the late Patron of the League, his Majesty King Edward VII. Probably no one in this country ever took such an interest in every charitable work for the benefit of the people; and of all the charities which received his patronage, in none did he take a greater interest than in the League of Mercy, of which he was Grand President from its foundation until his accession to the throne. And when he became King his interest in the League was unremitting. He was constantly asking about its prosperity, and expressed his great satisfaction at hearing that each year the League had collected more for the hospital funds than in the preceding year. He was sure all would regret very much that their Majesties had now been compelled to resign the positions which they had held so long as Grand President and Lady Grand President of the League. During the time they held those offices their Majesties worked unceasingly—(hear, hear)—and they left no stone unturned in their efforts to increase the prosperity of the League of Mercy, to which efforts much of the increase in the funds was due. Thanks were also due to their Majesties for having appointed their brother-in-law and sister-in-law, the Prince and Princess Alexander of Teck, to be Grand President and Lady Grand President. They had both worked for the League with great devotion, and their interest was proved by the success which attended their efforts in their several districts. The business of the League would be more immediately spoken to by Sir Henry Burdett and others; but he was happy to say that during the eleven years that the League had been in existence it had contributed £135,000 to the King Edward's Hospital Fund and £7,700 to the extra metropolitan hospitals. Those were very large sums, and they could not have been obtained without great self-sacrifice and very hard work by the various workers. And he thought he could safely say that those hospitals to which the League had contributed so largely had benefited very much in many ways by their connection with it; because the League had always sent their own inspectors to go carefully over those hospitals, and that inspection had been the means of doing them a great deal of good. He hoped the increased contributions could be continued, because he trusted that the League would be able to add to its own funds through their means. Though the present year had been nearly equal to last, it had not been quite so prosperous, and there were many reasons which, he hoped, adequately explained the cause. No doubt the General Election had something to do with it, because many meetings of the League had been put off in consequence. Also, he regretted to have to report that four or five of the League's workers had died during the year, and they were among the active workers. There had also been many resignations, which he did not like to have to speak about, and which he trusted were now at an end. He hoped more workers would be forthcoming to balance matters. He earnestly hoped that the workers in the League would redouble their efforts and see to it that never again would there be a year which was not better than the last one. He wished to take the opportunity of thanking several members of the Royal Family, who did really great work for the League. In this connection he specially mentioned the Duchess of Albany and the Princesses Victoria and Marie Louise of Schleswig-Holstein. One constantly saw their names in the papers in association with some good work, and he wished, on behalf of the League, to thank them for the way in which they had increased its prosperity. He desired to impress upon the meeting the concluding wish in the King's letter, that the energies of the League's workers should not cease; and reminded his hearers that the fact that the King was obliged to resign his Grand Presidency would not in any way affect those who were working, and he hoped all would put their shoulders to the wheel and do all that they possibly could. (Hear, hear.) He felt that gratitude was due not only to those whom he had named, but also to the secretaries; and he was pleased to see present his noble friend Lord Wolverton, who took such an interest in the work, and Sir William Collins, who did his best to promote the interests of the League. There was also his excellent friend Colonel Kempster, who had a grasp of the whole situation and did all he possibly could, and he would like to tender the thanks of the meeting to him and the staff who worked with him. (Applause.)

### Two Resolutions.

Mr. Carl Hentschel, C.C., moved: "That the Right Hon. Lord Farquhar, G.C.V.O., be appointed Chairman; Mr. Frederick Green, J.P., Deputy-Chairman; and Colonel H. B. Hamilton, Hon. Secretary, respectively." In doing so he wished to comment upon what the Chairman had said concerning the good work which the King expected to be done next year. As the Chairman had rightly said, the League had had rather a trying year, though in in his own district, that of the Strand, he had been lucky in exceeding the sum collected in the previous year—(applause)—and he wished to take the opportunity of tendering his warmest thanks to the members of the theatrical profession, who were always so ready to come forward and do work, without any stipulation, in the cause of charity. Though this year had been a trying one, next year would be a very important one, for it would be Coronation year, and it would, he thought, be a good thing if the President would call together all the Presidents and Lady Vice-Presidents to a Conference to consider whether the League should hold a Convention, in which all the Districts could participate, to do something in celebration of Coronation year. (Applause.)

Mr. Bertram S. Straus, in seconding, said it required no words from him to commend it to the meeting. The fact that this year had not been a good one for the League should, he thought, be a stimulus to put forth extra efforts in the coming year.

The resolution was carried.

Mr. Geo. Alexander proposed : " That seven representative members of the Executive Committee be appointed—The Right Hon. the Lord Farquhar, G.C.V.O., Colonel H. B. Hamilton, Mr. Fredk. Green, J.P., Mrs. R. L. Harrison, Mr. Edward Murray Ind, D.L., J.P., Lady Dimsdale, and Mr. Montagu Sharpe, D.L." He desired to offer the thanks of his profession for the kind words which had been uttered by Mr. Hentschel. He also had been fortunate this year in increasing his amount, and that had been in some measure contributed to by the personal work of many members of his profession. But he wished to remind the meeting that during these last years the South St. Pancras District of the League of Mercy had been almost asleep, and they had only had two years in which to wake them up. And, though it was a poor district, he was proud of its present year's work, and he hoped it would further increase. If the suggestion brought forward by Mr. Hentschel should take shape, it would be not only his personal pleasure, but also that of the members of his profession, to aid it in any way by their services. (Applause.)

The Rev. W. H. Hornby Steer, M.A., seconded the Resolution, and it was carried.

### The Treasurer's Statement.

Sir Henry Burdett, K.C.B. (Hon. Treasurer) said that, although the funds raised by the League this year were smaller than for last year, he did not consider the year just closing had been a bad one. Everything which could work against the raising of money for charities seemed to have combined this year to prevent any voluntary institution from thriving. It was the first time for 200 years that two elections had been held in this country in the same year; and as they occurred at the beginning and the end of the year, they cut into the golden days for the collection of money for charities. Those who were in the habit of collecting money from voluntary sources knew that the prosperous time for that purpose was from the middle of January to the middle of April, the first six weeks of that time being generally the most fruitful. For the end of the year, they began to harvest funds in mid-October, and worked till mid-December. This year that period had been almost barren, because it was impossible to devote one's energies to the raising of money in consequence of the election. In the middle of the year occurred the death of King Edward, an event of vast importance to the nation and Empire; and the shock of it seemed to paralyse the energies of many of his subjects. So their thanks were specially due to the workers of the League, because the results had been, on the whole, so good. The League had lost King George as Grand President and Queen Mary as Lady Grand President—a very great loss, because their Majesties believed in the doctrine of personal service, and they had practised it so steadily and continuously that every year those who had the pleasure of being at the garden party saw and knew that they were led by those who had the right spirit. They felt their own spirits raised, their energies increased, and their confidence in the League immeasurably strengthened in consequence of their Royal Highness' work. Surely it would stimulate them to remember all that our King and Queen did for the League during the ten years that it was under their leadership; and it should be upon those who had still to work and bear the burden to go forward with even greater energy, both in getting recruits and in raising money. The League had the countenance, support, and fellowship of members of the Royal Family to an extraordinary extent. Although most of those present realised how hard some members of the Royal Family had worked, few probably knew that there had been paid in by the districts presided over by members of the Royal Family £27,438. (Applause.) Princess Alexander and Prince Alexander of Teck had raised in their district £10,402; Princess Victoria of Schleswig-Holstein, £11,845; the Duchess of Albany, £2,872; and Princess Marie Louise, £2,319. (Applause.) Members of the League remained members because they believed in its principles, and because it was known to do an immense amount of good by gathering a harvest of small coins for the hospitals in a way that they could not be garnered otherwise. He was sure that the facts he had given would be a foreword for the League for 1911, and that everyone in the room and those whom they could speak to and influence would feel that they had a great responsibility and a greater privilege in determining that the next year should be the best year in the history of the League. Special efforts were needed in some of the larger districts. When the League was founded there used to be very big meetings, and out of the large number of people present at them the League gathered many new friends, who settled down steadily as members of the League. He threw out the hint now so that the Presidents might think it over. The central office would cordially co-operate with any district in the Metropolis which desired to have such a meeting, so that the area of operations could be extended by recruiting a large number of new members and associates in that way. It was the only way in which large numbers of people could be assembled to hear about the League. The total number of districts which had collected this year was ninety-five, as against ninety-eight last year. Two districts which had fallen out were Clapham and Willesden, while Harrow district had become extinct. The money paid in to the present date was £17,046, as against £17,783 last year. But the £17,000 this year was paid in from eighty-four districts, as against ninety districts last year, so that the average had been rather higher. A further £3,000 had been promised for this year, bringing the total collections for the year to over £20,000, as compared with £21,606 last year. The total amount distributed through the King's Fund to the hospitals was £135,000, and he hoped that this year there would be added £18,000, making the total amount given to the hospitals in London in eleven years £153,000. (Applause.) Presidents knew that grants were also made to country hospitals, and that system was being extended and the amount increased every year. Thus in 1910, £2,190 was voted to country districts, being an increase of £215 over the previous year. Altogether £7,660 had been given to country hospitals, making the grand total given to all hospitals by the League in eleven years £160,660. (Applause.)

COUNTRY DISTRICTS, LONDON AND LOCAL HOSPITALS.

He would like to say a word to those country districts of the League who sometimes thought that all or most of the money collected in their district should go to the local hospital. Patients were sent from those districts to London hospitals, and a census showed that 20 per cent. of the beds of the London hospitals were occupied by cases coming from such districts. The expenditure of the London hospitals, in round figures, was a million pounds a year, 20 per cent. of which was £200,000, and that might therefore be regarded as the value in money which the country districts received back in the shape of relief given to their patients. But the League had never raised more than from £20,000 to £25,000 in any one year, or only one-tenth of that £200,000 he had mentioned. He thought those figures should satisfy reasonable people that, as the first object of the League was to help the London hospitals, the claim of those hospitals upon the adjacent counties was a very onerous one, and no scheme had yet been submitted which would provide a solution by percentages. It could not be arranged that a definite percentage of 50 per cent. or 25 per cent. should be given to the hospitals in any one district; indeed, he did not think that would be the best way, even if a scheme could be devised, because applications were received for grants for all the hospitals which the Presidents and Lady Presidents in each country district thought worthy of support and entitled to a grant. Those applications were all gone into carefully, after the hospitals were inspected by the visitors and examined by the Distribution Committee. Such grants were made this year to a total of £2,190. He hoped these facts would be carefully thought over, and that anyone who had a scheme would communicate with him (Sir Henry). The object was to meet the wishes of those who were working for the League of Mercy, and to benefit all the hospitals, whether situated in London, or in the country districts where the League was at work. (Applause.) Sir Henry then read out the district contributions, indicating the increase or decrease in each case :

### YIELD OF EACH DISTRICT IN 1910.

S. Kensington £1,375, as against £1,536, a decrease of £161.
S. Paddington £1,108, as against £896, an increase of £212.
St. George's £1,005, as against £1,045, a decrease of £40.
Epsom—Esher £903, as against £674, an increase of £229.
Fulham £770, as against £864, a decrease of £103.
Hants £597, as against £686, a decrease of £89.
Berkshire £573, as against £688, a decrease of £115.
E. Marylebone £563, as against £519, an increase of £44.
W. Marylebone £552, as against £673, a decrease of £121.
Kingston—Surbiton £518, as against 517, an increase of £1.
Westminster £449, as against £519, a decrease £70.
Strand £433, as against £429, an increase of £4.
Chelsea £406, as against £280, an increase of £126.
Sevenoaks £403, as against £521, a decrease of 118.
N. Kensington £366, as against £311, an increase of £55.
S. St. Pancras £333, as against £209, an increase of £124.
City £325, as against £203, an increase of £122.
E. Sussex £319, as against £350, a decrease of £31.
Lewisham £319, as against £369, a decrease of £50.
Oxfordshire £313, as against £338, a decrease of £25.
Bucks £298, as against £354, a decrease of £56.
Hammersmith £288, as against £267, an increase of £21.
Tonbridge £285, as against £296, a decrease of £11.
N.E. Sussex £274, as against £240, an increase of £34.
Chertsey £264, as against £268, a decrease of £4.
Guildford £261, as against £257, an increase of £4.
N. St. Pancras £260, as against £304, a decrease of £44.
W. St. Pancras £258, as against £191, an increase of £67.
Luton £255, as against £209, an increase of £46.
Hornsey—Finchley £252, as against £335, a decrease of £83.
Mid-Essex £227, as against £293, a decrease of £66.
Hampstead £212, as against £270, a decrease of £58.
Richmond £206, as against £173, an increase of £33.
Dulwich—Norwood £197, as against £226, a decrease of £29.
Hertford £196, as against £191, in increase of £5.
Epping £195, as against £197, a decrease of £2.
Eastbourne £184, as against £203, a decrease of £19.
Battersea £175, as against £161, an increase of £14.
Maldon £166, as against £140, an increase of £26.
Ashford £164, as against £142, an increase of £22.
Eye £162, as against £193, a decrease of £31.
North Hackney £157, as against £135, an increase of £22.
Lambeth (Brixton) £159, as against £202, a decrease of £43.
S.E. Essex £154, as against £158, a decrease of £4.
Deptford—Brockley £149, as against £294, a decrease of £145.
Ealing £146, as against £298, a decrease of £152.
Horsham £145, as against £166, a decrease of £21.
Medway £144, as against £138, an increase of £6.
Woolwich £141, as against £167, a decrease of £26.
Brentford £138, as against £235, a decrease of £97.
Hitchin £135, as against £146, a decrease of £11.
Reigate £131, as against £121, an increase of £10.
Southwark—Bermondsey £128, as against £135, a decrease of £7.
Romford £126, as against £133, a decrease of £7.
Greenwich £123, as against £114, an increase of £9.
Stowmarket £116, as against £123, a decrease of £7.
Uxbridge £109, as against £160, a decrease of £51.
East Grinstead £108, as against £132, a decrease of £24.
Dartford £106, as against £25, an increase of £81.
Watford £102, as against £89, an increase of £13.
Mile End £86, as against £113, a decrease of £27.
Enfield £81, as against £100, a decrease of £19.
Lewes £79, as against £82, a decrease of £3.
Croydon £73, as against £135, a decrease of £62.
Gravesend £72, as against £107, a decrease of £35.
Brighton—Hove £70, as against £92, a decrease of £22.
Whitechapel £69, as against £69, no change.
West Islington £66, as against £75, a decrease of £9.
Poplar—Limehouse £61, as against £61, no change.
Streatham £60, as against £66, a decrease of £6.
Harwich £55, as against £58, a decrease of £3.
N. Camberwell £54, as against £57, a decrease of £3.
Hythe £54, as against £72, a decrease of £18.
Biggleswade £51, as against £62, a decrease of £11.
Sudbury £49, as against £43, an increase of £6.
Rochester £46, as against £43, an increase of £3.
St. Albans £45, as against £40, an increase of £5.
Wimbledon £45, as against £73, a decrease of £28.
Faversham £42, as against £39, an increase of £3.
E. St. Pancras £41, as against £32, an increase of £9.
Thanet £41, as against £88, a decrease of £47.
Bethnal Green £38, as against £54, a decrease of £16.
Finsbury, East, £36, as against £40, a decrease of £4.
Worthing £37, as against £47, a decrease of £10.
Lambeth—Kennington £30, as against £43, a decrease of £13.
Walthamstow £27, as against £27, no change.
Finsbury, Central, £26, as against £123, a decrease of £97.
Holborn £26, as against £49, a decrease of £23.
Lowestoft £20, as against £44, a decrease of £24.
Peckham £15, as against £29, a decrease of £14.
N. Paddington £13, as against £15, a decrease of £2.
Saffron Walden £10, as against £21, a decrease of £11.
W. Southwark £9, as against £24, a decrease of £15.
Chichester £7, as against £7, no change.

The return from Colchester, which last year was £63, is unascertainable at the moment owing to the sudden death of the husband of the Lady-President.

### No Ground for Discouragement.

Sir Henry Burdett's concluding word was that the Presidents and their workers must not be discouraged because there had been a falling-off in some districts, for it would be seen that where there were increases some of them were far larger than was the amount of the falling-off in any district which had not quite reached last year's figure. All had in fact done remarkably well in the most difficult year of the League's existence.

### The Lady President for Westminster.

Her Grace the Duchess of Somerset said she had been asked to say a few words as to the work of the district of London. She desired to commence by congratulating the workers in the different districts for the splendid way in which they had worked during the year, which, it was well known, had been a difficult year. She would never have dared to criticise the work of any district but her own, but she felt that the League had worked under considerable difficulty this year in collecting and in maintaining interest, and she wished to say a word or two which would be of some assistance to the workers in the other districts of London. First, in regard to organisation. The importance of organisation had been put closely before them during the last few weeks, and if the organisation of the League were better in different districts, her Grace thought more money would be collected for the League, in which all felt such a deep interest. One suggestion she had to make was that there should be a closer touch with the workers. She did not mean that everyone should give entertainments : that would be impossible. But Sub-Committees could be formed, who would collect a few workers, and once a year each Vice-President could collect their own workers and have a little quiet talk as to how best to deal with different streets and localities. A splendid example had been set by the Royal Family in the work they had done for the League. The Presidents were very much indebted to their Majesties the King and Queen for the close touch they had kept with the League of Mercy during the past ten years. (Hear, hear.) Though she had it in mind to cease her activities in certain charities, because they seemed to tax her strength, she had felt much encouraged by the splendid example set by the King and Queen and the other members of the Royal Family, and she hoped now to continue to work the district of Westminster for the League of Mercy certainly for some time longer, and, she hoped, with more success than in the past. (Applause.) She felt it was a great privilege to work. Amid the technical matters which were put forward at a meeting like the present one sometimes forgot what a privilege it was to work in the cause of the sick and suffering who lay in every quarter of London, in whatever direction one moved or had his or her sphere. The work of the London hospitals had been a very splendid one in the past, and she would much regret if the enterprise and generosity of the public were ever hampered or interfered with by the hospitals being placed under State protection. She hoped such a day would be far distant. Her great wish was that the workers for the League would feel encouraged to work still more strenuously in the future than in the past. She had been asked to say one word on that occasion by those who felt that the work for the League should be entirely kept for the cause of the sick and suffering in the hospitals, and she would be glad if an assurance could be given on that point. She did not say it on her own behalf, but she had been asked to bring the point very strenuously before the meeting. With such an assurance she felt confident that the workers would go forward and do better work for the League even than in the past. (Applause.)

### The Hon. Secretary's Statement.

Sir W. J. Collins, M.D., on behalf of his colleagues, the hon. secretaries, had a few remarks to add. The small subscribers had come forward well in the past year. The grants made by the League to county hospitals in the year 1910 show an increase of 11 per cent. over those made in 1909.

The Grand President and Lady Grand President have, during the last twelve months, been pleased to appoint the following Presidents and Lady Presidents in the districts named :—

London Districts.—Chelsea, Mrs. William Playfair; Clapham, Mrs. Mateo Clark; Hackney, North, Miss Constance G. Johnson; Fulham, Mr. Philip F. Walker; Hammersmith, Mrs. Rodwell; Hampstead, Mrs. Metzler; Marylebone, East, Sir Herbert Beerbohm Tree; Woolwich, Major Adam; Whitechapel, The Right Hon. W. Ellison Macartney.

Country Districts.—Dartford, Mrs. Jay and Mr. John Grant; Harrow, Mr. J. N. Stuart; Maldon, Mr. and Mrs. Thos. Philip Price; Mid-Essex, The Hon. Mrs. Greville; Richmond, The Right Hon. The Lord Farquhar, G.C.V.O.; Saffron Walden, Mr. R. J. Bliss Howard and The Hon. Mrs. Howard; Hythe, Miss Campbell of Auchmannoch.

His Serene Highness Prince Alexander (Grand President) awarded the Order of Mercy, at St. James's Palace, in July, to forty-eight ladies and gentlemen connected with the League. The total award of Orders was now 430.

H.R.H. Princess Alexander of Teck held two meetings in December at Broom Hill, in connection with the districts of Epsom-Esher with Godstone-Caterham, and Kingston-Surbiton with Wandsworth-Putney.

Numerous drawing-room and other meetings have been held, at which addresses were given by the honorary officers and others connected with the League.

### List of Awards to County Hospitals.

With the sanction of H.S.H. Prince Alexander of Teck (Grand President of the League of Mercy), the following grants have been made to Extra-Metropolitan hospitals by the League :—

Berkshire.—Royal Berkshire Hospital, Reading, £200, and £100 conditional; King Edward VII. Hospital and Dispensary, £30; Coldash Cottage Hospital, Newbury, £10; Faringdon Cottage Hospital, £10.

Buckinghamshire.—Royal Bucks Hospital, Aylesbury, £50; Marlow Cottage Hospital, £10; Iver Langley and Denham Cottage Hospital, £5; Chalfont St. Peter's Cottage Hospital, £10; Chesham Cottage Hospital, £10.

Essex.—Essex County Hospital, £35; Chelmsford and Essex Hospital and Infirmary, £40; Romford Victoria Cottage Hospital, £15; Victoria Hospital, Southend-on-Sea, £20; Brentwood Cottage Hospital, £10; Buckhurst Hill Village Hospital, £15; Eden Cottage Hospital, Hatfield Broad Oak, £10; Saffron Walden Hospital, £10.

Hampshire.—Royal South Hants Hospital, Southampton, £150; Royal Hants County Hospital, Winchester, £150; Royal Portsea, Portsmouth and Gosport Hospital, £100; Fleet Cottage Hospital, £10.

Hertfordshire.—Hertford County Hospital and General Infirmary, £50; Watford District Hospital, £25.

Kent.—West Kent General Hospital, Maidstone, £25; Gravesend Hospital, £30; General Kent and Canterbury Hospital, £30; Tunbridge Wells General Hospital, £20; Folkestone Victoria Cottage Hospital, £20; St. Bartholomew's Hospital, Rochester, £20; Ashford Cottage Hospital, £15; Sevenoaks Hospital for Children with Hip Disease, £20; Beckenham Cottage Hospital, £20; Bromley Cottage Hospital, £20; Chislehurst and Cray Cottage Hospital, £10; General Hospital and Seamen's Infirmary, Ramsgate, £15;

Margate Victoria Home for Children, £15; Tunbridge Wells Homœopathic Hospital, £10; Tonbridge Queen Victoria Cottage Hospital, £5.

Middlesex.—Enfield Cottage Hospital, £15; Hounslow Hospital, £20; Hanwell Cottage Hospital, £10; St. John's Hospital, Twickenham, £10; Teddington and Hampton Wick Cottage Hospital, £10.

Oxfordshire.—Radcliffe Infirmary, Oxford, £125; Horton Infirmary, Banbury, £30; The Eye Hospital, Oxford, £20.

Suffolk.—West Suffolk General Hospital, Bury St. Edmunds, £30; East Suffolk and Ipswich Hospital, £50; St. Leonard's Hospital, Sudbury, £10; Southwold Cottage Hospital, £5.

Surrey.—Royal Surrey County Hospital, £75; Croydon General Hospital, £30; Egham Cottage Hospital, £15; Surbiton Cottage Hospital, £10; Woking, Victoria Cottage Hospital, £10; Reigate and Redhill Cottage Hospital, £20; Caterham Cottage Hospital, £10; Kingston Victoria Hospital, £10; Leatherhead Victoria Memorial Cottage Hospital, £5; Purley Cottage Hospital, £10; Thames Ditton Cottage Hospital, £5; Carshalton District Hospital, £10; Surgical Home for Boys, Banstead, £10.

Sussex.—Hastings, St. Leonards and East Sussex Hospital, £150; Royal Alexandra Hospital for Children, Brighton, £25; Haywards Heath Cottage Hospital, £15; Eastbourne Princess Alice Memorial Hospital, £15; Buchanan Hospital, St. Leonards, £10; St. Bernard's Home for Gentlewomen, £10; Hastings and St. Leonards Home for Invalid Gentlewomen, £10; Lewes Dispensary and Infirmary, £15; Brighton and Hove Hospital for Women, £10; Horsham Cottage Hospital, £10; Leaf Homœopathic Cottage Hospital, £10; Uckfield Cottage Hospital, £5.—Total, £2,190.

H.H. Princess Victoria of Schleswig-Holstein has paid visits of inspection to hospitals during the past year in the counties over which she presides.

Sir William Collins gave instances of quite considerable sums of money having been collected in single pennies—for example, £20 11s. 11d. was collected in 4,943 individual penny subscriptions. (Applause.) In answer to the observations which had been made by the Duchess of Somerset, the League relied on the assurance given them by the King's Hospital Fund, that the moneys contributed to the hospitals through its agency went exclusively to the relief of the sick poor; and that principle, he believed, would be continued. An inquiry would be held in regard to the out-patient departments of the London hospitals to ascertain whether they were being made use of by persons who were ineligible for them, and to secure that suitable and adequate accommodation was provided for those who did make use of them. (Hear, hear.) He was informed by the hon. treasurer that Lady Chesterfield had to be congratulated on the fact that South Kensington had given the largest amount collected this year. (Applause.) As he was conversant with the working of the League, he was amazed that this year had come out as well as it had. Those who worked from the start of the League knew what intense interest and personal enthusiasm his late Majesty King Edward put into the launching of the League. The workers felt that unfortunate events might follow the blow sustained by his death; but he was glad to know that his present Majesty would continue to take an unabated interest in the work, though not as Grand President. He hoped that next year there would be a larger record than ever before, and thus commemorate the Coronation year. (Applause.)

### Votes of Thanks.

Sir Wm. Bull, M.P., proposed "That the authorities of Middlesex Hospital be thanked for their kindness in granting the use of the board-room for this meeting, and took the opportunity of expressing his condolence with the authorities of that hospital in the loss they had recently sustained by the death of H.S.H. Prince Francis of Teck.

Mr. H. W. Keay, J.P., seconded, and it was carried.

On the proposition of Mr. Carl Stettauer, seconded by Dr. Scott, Lord Farquhar was cordially thanked for occupying the chair and for his great work in connection with the League.

The Chairman acknowledged the vote, and the meeting concluded.

### The Grand President.

Prince Alexander of Teck held an informal reception after the meeting, and displayed much interest in the proceedings.

